# Effectiveness of platelet-rich plasma in pain management of osteoarthritis with developmental dysplasia of the hip: a double-blind, randomized controlled trial

**DOI:** 10.1093/jhps/hnaf008

**Published:** 2025-03-05

**Authors:** Yusuke Okanoue, Masahiko Ikeuchi, Junpei Dan, Yuki Teranishi

**Affiliations:** Department of Orthopaedic Surgery, Kochi Medical School, Nankoku, Japan; Department of Orthopaedic Surgery, Kochi Medical School, Nankoku, Japan; Department of Orthopaedic Surgery, Kochi Medical School, Nankoku, Japan; Department of Orthopaedic Surgery, Kochi Medical School, Nankoku, Japan

## Abstract

This study evaluates the efficacy of administering platelet-rich plasma (PRP) compared to hyaluronic acid (HA) for pain management hip osteoarthritis (OA) secondary to developmental dysplasia of the hip (DDH). It highlights PRP treatment as a slightly more effective or equivalent treatment for reducing hip pain in such cases. From 2019 to 2021, a double-blind, randomized controlled trial was conducted with 42 patients who consented to participate. They were divided into two groups: one receiving intra-articular PRP injections and the other HA injections. The primary focus of the study was pain relief, measured using the pain-Visual Analogue Scale (VAS) and Western Ontario and McMaster Universities Osteoarthritis Index (WOMAC)-pain scores over a 24-week period. Functionality was assessed as a secondary outcome. The results showed significant pain reduction in both PRP and HA groups compared to their baseline pain levels. Notably, the PRP treatment group exhibited a marginally higher improvement in pain-VAS scores (38.5) than the HA group (18.7; *P* = .041). However, the difference in WOMAC-pain scores between the groups was not statistically significant (4.3 for PRP vs. 2.9 for HA; *P* = .245). The Kellgren–Lawrence grade was the only factor significantly associated with the improvement in pain-VAS scores within the PRP group. The study finds that PRP treatment is at least as effective as HA treatment in reducing hip pain for OA secondary to DDH. Treatment with PRP showed notably better pain-VAS scores compared to HA, highlighting its potential. Therefore, intra-articular PRP injections are a viable alternative to HA for effectively reducing pain in OA secondary to DDH.

## Introduction

Hip osteoarthritis (OA) is one of the most common joint diseases. The worldwide prevalence of hip OA was estimated at 40 million people and the global incidence at 2 million people [1]. Developmental dysplasia of the hip (DDH) is characterized by morphological deformation (insufficient acetabular coverage of the femoral head, shallow acetabulum, excessive femoral anteversion, coxa valga, and shortening of the femoral neck). It is the main cause of secondary hip OA due to a decrease in the contact area of the articular surface, abnormal distribution of stress to the articular cartilage, and labral tear. According to previous reports, DDH is involved in 20%–40% of patients with hip OA [2]. Management for DDH includes various conservative as well as surgical treatments. Conservative therapies such as weight loss, intra-articular injections, and physiotherapy have been reported to relieve pain in OA patients with DDH and slow the progression of OA [3]. However, since their efficacy is limited, there are many cases of hip OA that require surgical treatments, such as periacetabular osteotomy (PAO) [4], rotational acetabular osteotomy (RAO) [5], and total hip arthroplasty (THA).

Recently, platelet-rich plasma (PRP) intra-articular injection has been attracting attention as a new conservative treatment for hip OA. PRP is a product derived from autologous peripheral blood containing various growth factors and adhesion factors, which are essential for tissue repair. Its injection into injured tissues exhibits an anti-inflammatory action through its effect on the Nuclear factor kappa B (NF-κB) signal transduction pathways of synovial cells, macrophages, cartilage cells, and the like [6–10]. In addition, because the treatment uses only the patient’s blood components, it has the great advantage of not being prone to triggering an immune response. However, the results of comparisons with intra-articular injection of hyaluronic acid (HA) and steroids were inconsistent in previous reports [11–13], and there is still controversy regarding PRP intra-articular injection in hip OA. In addition, there have not been any reports on PRP intra-articular injection for hip OA with DDH, and the effect of the mechanical stresses in the morphological deformations on the pain-relieving effects of PRP intra-articular injection is still unclear.

This randomized controlled trial (RCT) examined the efficacy of administering PRP compared with HA treatment of the symptoms of hip OA secondary to DDH. Factors associated with pain improvement were also assessed in the PRP group.

## Materials and methods

### Study design

This study is a double-blind, randomized, controlled trial. It was approved by the Specially Certified Committee for Regenerative Medicine (approval number PB6180006) and authorized by the Ministry of Health, Labour, and Welfare of Japan. All patients examined in the study provided written informed consent.

### Patient selection and treatment

The participants were patients with symptomatic hip OA with the following characteristics: (I) the chief complaint was hip pain and they met the American College of Rheumatology criteria; (II) between 20 and 80 years old at the time informed consent was obtained, (III) the hip pain had continued for ≥3 months, with a visual analog scale (VAS) of ≥30 mm, (IV) a Kellgren–Lawrence (K–L) grade of 1–3 on plain X-ray, (V) body mass index (BMI) of ≤30 kg/m^2^, and (VI) requiring treatment on one side only. The exclusion criteria were as follows: (I) patients exhibiting polyarthralgia, (II) femoral head necrosis, (III) infection, (IV) autoimmune disease (various connective tissue disorders such as rheumatoid arthritis and systemic lupus erythematosus), (V) underlying blood disorders (coagulation/fibrinolytic system abnormalities such as haemophilia), and (VI) using anticoagulants.

Between February 2019 and March 2021, 42 patients met the inclusion criteria and gave informed consent to participate in this study; they were randomized into two groups via an electronic randomization process. One group received intra-articular PRP, and the other group received intra-articular HA. For blinding purposes, the study staff performed the randomization, the principal investigator (Y.O.) performed the injection, and an evaluating physician (J.D. and Y.T.) performed the evaluation. The patients and evaluating physicians were not informed of their assignments. In addition, 90 ml of blood was sampled from all patients, which was subsequently used only in the PRP group.

**Figure 1.**
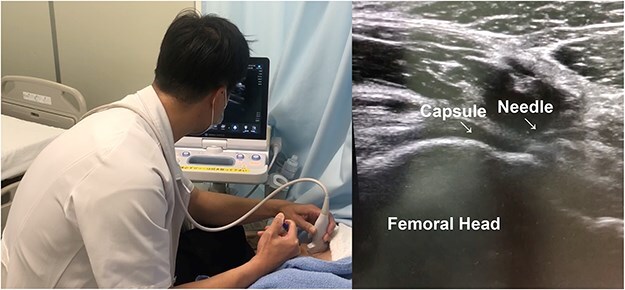
Ultrasound-guided PRP or HA injection for the hip joint using the anterosuperior parasagittal approach.

Every 2 weeks, a total of three ultrasound-guided intra-articular injections of the hip joint were performed by an investigator not involved in the evaluation. The intra-articular injections were performed by inserting a 22-gauge, 90-mm Cathelin needle (Terumo Corporation, Tokyo, Japan) under sterile conditions. The PRP and HA were evenly dispersed inside the hip joint by injecting 1 ml PRP or 2.5 ml HA into the base of the femoral neck with an anterosuperior parasagittal approach following local anaesthesia with 3 ml 1% lidocaine. Proper needle position was confirmed by direct visualization of the injected PRP/HA fluid (Fig. 1).

The HA used in this study was a commercially available product, Artz Dispo® (Seikagaku Corporation, Tokyo, Japan). It is a sterile, nonpyrogenic, viscoelastic solution of highly purified, natural sodium hyaluronate derived from rooster combs, with a molecular weight of approximately 900 000–1 200 000 Da. Each 2.5-ml pre-filled syringe contains 25 mg of sodium hyaluronate.

### PRP preparation

PRP preparation was performed by a Specific Processed Cell Manufacturer (FUJISOFT Cell Processing Center, Tokyo, Japan). Peripheral blood samples (90 ml), including 7% sodium citrate, were sampled. The peripheral blood was centrifuged at 200 G for 15 min, and the plasma was recovered after avoiding the buffy coat laminated on the interface between the plasma and the erythrocytes. The plasma was sampled (0.5 ml) for measurement of the platelet count. The remaining plasma was centrifuged for 15 min at 1200 G, the excess platelet-poor plasma (PPP) was removed, and 4.5 ml of 20-fold concentrated leucocyte-poor PRP (LP-PRP) was prepared. The refined PRP was classified as P2-x-Bβ per the PAW classification [6]. The PRP (3 ml) was aliquoted into three vials (1 ml each) and cryopreserved. In sterility testing, 1 ml of the PRP was used, and 0.5 ml of the PRP was used for ELISA to evaluate the epidermal growth factor (EGF). For platelet count measurement, 0.5 ml of the PPP was used. The platelet count in the PRP was calculated by subtracting the platelet count in the PPP from the platelet count in the plasma. After confirming that the result of sterility testing was negative, the PRP was cold transported to the hospital and cryopreserved after arrival until the time of use.

### Outcome measurement

The patients were clinically evaluated using subjective and objective evaluations at baseline, at Weeks 2, 4, 8, 12, 18, and 24 after treatment.

The primary outcomes were improvement in the pain-VAS while walking and Western Ontario and McMaster Universities Osteoarthritis Index (WOMAC)-pain scores at 24 weeks. For the secondary outcomes, the WOMAC total score, Harris hip score (HHS), and Oxford hip score (OHS), as well as the correlation between the degree of improvement in the pain-VAS and WOMAC-pain scores with associated factors [KL-grade, lateral centre edge angle (LCEA), age, BMI, and EGF], were evaluated. Moreover, the patients were divided into two groups [above and below the minimal clinically important difference (MCID)] for the pain-VAS and WOMAC-pain scores [7].

### Sample size and statistical analysis

When significance testing was performed using the mean between the two groups using the *t*-test on the premise that a difference of at least 50% in other clinical studies was observed in pain-VAS scores after 24 weeks and the significance level was assumed to be 5%, the number of cases needed to perform significance testing at a test power of 80% was deemed to be 16.6. Given that the statistically necessary number of patients was *n* = 17, we added *n* = 3 as dropout cases and set the number of patients at *n* = 20 for each group.

An independent *t*-test and chi-squared test were used to compare the demographic data of the two groups. Two-way repeated-measures analysis of variance (ANOVA) was used to analyse the group × time interaction. In addition, an independent *t*-test was used for between-group comparisons and a paired *t*-test was used for pre-post mean difference comparisons (baseline and 6 and 24 weeks). To compensate for multiple comparisons, Bonferroni corrections were applied, and *P*-values were derived from these corrections. The bivariate Pearson correlation test was used to analyse the correlation between the associated factors and the clinical evaluations of the patients. For all results, *P* < .05 was treated as a statistically significant difference. All analyses were performed using the SPSS software v.26.0 for Windows (IBM Corporation, New York, USA).

## Results

The mean age of the 42 patients in the initial study stage was 55.5 (29–74) years. All were females, with a mean BMI of 22.4 (16–29). The mean EGF levels were 4550.4 (1639–8097) pg/ml in the PRP group.

**Figure 2.**
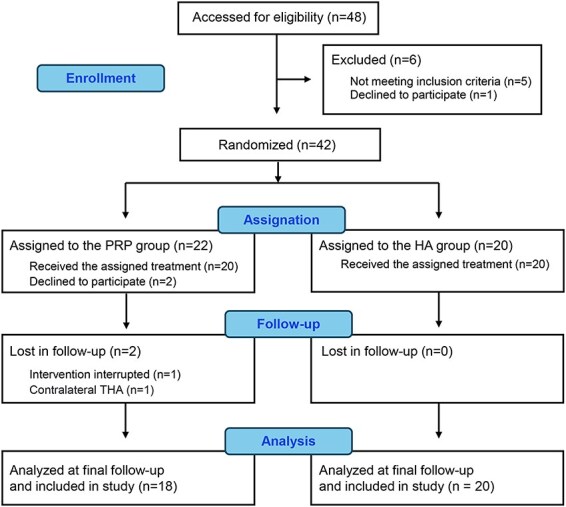
Patient recruitment flowchart.

A total of four patients (PRP group *n* = 4) were excluded from the final analysis for reasons such as revocation of consent, poor management, and requiring other joint surgery. The final study population included 18 patients in the PRP group and 20 patients in the HA group (Fig. 2). There were no significant differences between the two groups with regard to age, BMI, Kellgren–Lawrence (K–L) grade of the OA, pain-VAS and WOMAC scores, HSS, and OHS. (Table 1).

**Table 1.** Demographic data before treatment.

	PRP group	HA group	*P*-value
Patients (*n*)	18	20	
Age (years)	56.4 (39–75)	55.6 (29–70)	.802
BMI (kg/m^2^)	22.3 (19–27)	22.8 (15–29)	.687
LCEA (°)	12.2 (−7 to 24)	11.5 (4–23)	.724
KL grade (*n*)			.402
Grade 1	3	2	
Grade 2	9	7	
Grade 3	6	11	
Pain-VAS (0–100)	61.9 ± 5.7	54.8 ± 5.4	.276
WOMAC scores			
Pain (0–20)	8.3 ± 0.9	8.3 ± 0.9	.851
Function (0–68)	22.8 ± 3.8	18.6 ± 0.6	.593
Stiffness (0–8)	3.9 ± 0.4	3.4 ± 0.6	.409
Total (0–96)	35.0 ± 2.6	30.2 ± 3.9	.654
HHS	59.0 ± 2.6	58.8 ± 2.8	.965
OHS	29.8 ± 2.7	26.5 ± 1.8	.478
EGF (pg/ml)	1929 (1639–8097)		N/A

**Figure 3.**
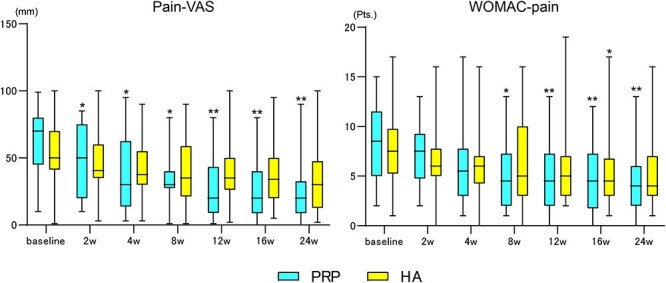
Box-and-whisker plots showing the treatment effect of PRP and HA over time. There was a significant improvement in the pain-VAS score compared with the baseline score after treatment (*P < .05, **P < .01 vs. baseline.) The two-way repeated-measures ANOVA test revealed that repetitive measurements of the pain-VAS and WOMAC-pain scores exhibited no significant differences between the two groups (*P* = .099 and *P* = .720, respectively).

For the primary outcomes (pain-VAS and WOMAC-pain score), the PRP group demonstrated significant improvements from baseline in pain-VAS at Weeks 4, 8, 12, 16, and 24 and in WOMAC-pain score at Weeks 8, 12, 16, and 24, indicating that the pain improvement associated with PRP persisted throughout the 24-week observation period. In contrast, the HA group showed a significant improvement from baseline in WOMAC-pain score at Week 16 only. The improvement in the pain-VAS scores at 24 weeks was significantly higher in the PRP group than in the HA group (38.5 vs. 18.7; *P* = .041). On the contrary, the improvement in the WOMAC-pain scores at 24 weeks was not significant in the two groups (4.3 vs. 2.9; *P* = .245). Regarding the secondary outcomes, the PRP group exhibited significant improvements in the WOMAC stiffness scores at 24 weeks compared with the HA group (2.6 vs. 1.4; *P* = .045), whereas no significant differences were exhibited in other outcomes (Table 2). The two-way repeated-measures ANOVA test revealed that repetitive measurements of the pain-VAS and WOMAC-pain scores exhibited no significant differences between the two groups (*P* = .099 and *P* = .720, respectively; Fig. 3). Regarding the secondary outcomes, the PRP group exhibited significant improvements compared with baseline in the WOMAC stiffness score, WOMAC physical function score, HHS, and OHS, whereas, the HA group exhibited significant improvements compared with baseline in the WOMAC stiffness score and HHS (Table 3). The proportion of patients whose scores were above the MCID for the WOMAC-pain scores was significantly higher in the PRP group than in the HA group at 24 weeks (94% vs. 55%; *P* = .004), whereas no significant differences were observed in the pain-VAS score (89% vs. 75%; *P* = .274).

**Table 2.** Improvement in study endpoints from baseline at each follow-up visit.

	PRP	HA	Effect size (95%CI)	*P*-value
Primary outcome				
Pain-VAS				
8 weeks	28.2 ± 6.5	14.1 ± 6.6	0.495 (−0.155 to 1.139)	.136
24 weeks	38.5 ± 6.2	18.7 ± 6.9	0.688 (0.270 to 1.339)	.041*
WOMAC-pain score				
8 weeks	3.0 ± 1.0	1.9 ± 1.0	0.249 (−0.392 to 0.887)	.448
24 weeks	4.3 ± 0.7	2.9 ± 1.0	0.384 (−0.262 to 1.024)	0.245
Secondary outcome				
WOMAC stiffness score				
8 weeks	1.5 ± 0.5	0.8 ± 0.4	0.350 (−0.294 to 0.990)	.288
24 weeks	2.6 ± 0.4	1.4 ± 0.4	0.675 (0.015 to 1.326)	.045*
WOMAC function score				
8 weeks	9.7 ± 2.9	4.5 ± 2.7	0.431 (−0.216 to 1.073)	.193
24 weeks	12.0 ± 2.9	5.3 ± 3.4	0.487 (−0.163 to 1.130)	.143
WOMAC total score				
8 weeks	14.2 ± 4.2	7.2 ± 3.9	0.403 (−0.243 to 1.044)	.223
24 weeks	18.8 ± 3.8	9.5 ± 4.5	0.513 (−0.138 to 1.157)	.123
HHS				
8 weeks	10.7 ± 2.6	10.8 ± 2.8	0.011 (−0.626 to 0.648)	.973
24 weeks	16.3 ± 2.9	14.1 ± 2.8	0.183 (−0.447 to 0.820)	.576
OHS				
8 weeks	7.5 ± 2.3	3.2 ± 1.4	0.551 (−0.102 to 1.196)	.099
24 weeks	9.1 ± 2.4	3.8 ± 1.6	0.613 (−0.043 to 1.262)	.067

Data are presented as mean ± standard error.

* *P* < .05: paired *t*-test in each group.

**Table 3.** All scores at baseline and follow-up in the treatment groups.

		Baseline	Treatment 2 (Week 2)	Treatment 3 (Week 4)	8 weeks	12 weeks	16 weeks	24 weeks	*P*-value (group × time)
Pain-VAS	PRP	61.9 ± 5.7	49.2 ± 5.8*	37.9 ± 6.6*	33.7 ± 4.7*	27.5 ± 5.5**	24.7 ± 4.7**	23.4 ± 5.0**	.099
	HA	54.8 ± 6.4	47.0 ± 5.5	43.2 ± 4.9	40.7 ± 5.1	41.1 ± 6.0	40.0 ± 5.8	36.2 ± 6.6	
WOMAC									
Pain	PRP	8.3 ± 0.9	7.2 ± 0.7	6.3 ± 1.0	5.3 ± 0.9*	4.8 ± 0.8**	4.6 ± 0.8**	4.1 ± 0.7**	.720
	HA	8.3 ± 0.9	6.8 ± 0.9	6.3 ± 0.8	6.4 ± 1.0	6.0 ± 1.0	5.6 ± 0.9*	5.4 ± 0.9	
Stiffness	PRP	3.9 ± 0.4	3.3 ± 0.4	2.9 ± 0.5	2.4 ± 0.4	2.2 ± 0.4**	2.1 ± 0.4*	1.3 ± 0.3**	.234
	HA	3.4 ± 0.5	2.8 ± 0.5	2.6 ± 0.5	2.6 ± 0.5	2.5 ± 0.5	2.3 ± 0.4	2.0 ± 0.4*	
Physical function	PRP	22.8 ± 3.8	22.1 ± 3.5	19.1 ± 4.1	13.1 ± 2.7*	12.3 ± 3.1*	10.7 ± 2.8**	10.8 ± 3.1**	.075
	HA	18.6 ± 2.6	15.4 ± 2.4	14.1 ± 1.9	14.1 ± 2.7	14.1 ± 2.9	13.0 ± 2.7	13.3 ± 3.2	
HHS	PRP	59.0 ± 2.6	65.2 ± 2.8	67.2 ± 3.6	69.7 ± 3.1**	69.6 ± 3.1*	74.1 ± 2.8**	75.3 ± 2.3**	.721
	HA	58.8 ± 2.8	62.3 ± 2.6	66.6 ± 2.4	69.6 ± 2.6*	67.8 ± 2.7	68.9 ± 2.7*	72.8 ± 3.0**	
OHS	PRP	29.8 ± 2.7	24.9 ± 1.7	24.1 ± 2.1	22.3 ± 1.8*	21.2 ± 1.5**	20.7 ± 1.7**	20.7 ± 1.8*	.111
	HA	26.5 ± 1.8	23.8 ± 1.8	22.9 ± 1.4	23.3 ± 1.9	23.3 ± 1.9	23.1 ± 1.7	22.7 ± 1.9	

Data are presented as mean ± standard error.

* *P* < .05,

** *P* < .01: paired *t*-test for difference from baseline in each group. *P*-value: two-way repeated-measures ANOVA used to analyse the group and time interaction (Greenhouse–Geisser correction).

Regarding the correlation between the pain-VAS improvement rate and the associated factors (K–L grade, CE angle, age, BMI, and EGF) in the PRP group, the K–L grade was the only factor associated with the pain-VAS improvement (Table 4). In the subsequent *post hoc* subanalysis stratified by the K–L grade, we found no significant differences in the degree of pain-VAS improvement between the PRP and HA groups within each K–L grade.

**Table 4.** Regarding the correlation between the pain-VAS and WOMAC-pain score improvement rates and the associated factors.

	Pain-VAS	WOMAC-pain score
Age	−0.255	−0.114
BMI	−0.017	0.393
LCEA	0.260	0.186
K–L grade	−0.450*	−0.282
EGF	0.382	0.287

The bivariate Pearson correlation test.

* *P* < .05.

Bacterial culture tests of the prepared PRP were negative for all specimens. Although the 19 patients underwent 55 intra-articular injections of PRP, no serious adverse events were observed. Some patients complained of injection pain during the procedure which disappeared within 24 h.

## Discussion

To the best of our knowledge, this study is the first double-blind RCT, wherein all the patients had hip OA with DDH, demonstrating that PRP significantly reduced hip pain regardless of the degree of acetabular dysplasia. The PRP group demonstrated significant improvements from baseline in pain-VAS at 4, 8, 12, 16, and 24 weeks and in WOMAC-pain score at 8, 12, 16, and 24 weeks, indicating that the pain improvement associated with PRP persisted throughout the 24-week observation period. In contrast, the HA group showed a significant improvement from baseline in WOMAC-pain score at 16 weeks only. The improvement in the pain-VAS scores at 24 weeks was significantly higher in the PRP group than in the HA group (*P* = .041). Moreover, The proportion of patients whose scores were above the MCID for the WOMAC-pain scores was significantly higher in the PRP group than in the HA group at 24 weeks (94% vs. 55%; *P* = .004). Nevertheless, it should be emphasized that there was an imbalance in the baseline severity of joint degeneration between the two groups, with a higher proportion of patients classified as K–L Grade 3 in the HA group than in the PRP group. This discrepancy may partly explain the differences in outcomes, suggesting that factors other than the treatment itself could have influenced the observed results. Based on our findings, no significant association was found between the degree of dysplasia and the improvement in pain-VAS scores within the PRP group. Therefore, it was apparent that administration of PRP significantly reduced hip pain even in the presence of abnormal joint stress and associated instability caused by the incongruence between the femoral head and the acetabulum. It is thought that a major causal factor of this is an anti-inflammatory action playing an important role in the pain-relieving effects of PRP on OA. Moreover, when evaluated solely within the PRP group, the pain-relieving effect appeared to be influenced by the severity of OA. However, in a *post hoc* subanalysis stratified by the K–L grade, although the sample size was limited, no significant differences in pain-VAS improvement were found between the PRP and control groups within each grade. Thus, this association does not apply when comparing PRP and HA. Nonetheless, further large-scale studies are warranted to clarify the influence of OA severity.

It is thought that the anti-inflammatory action of PRP plays a significant role in its pain-relieving effects on OA. PRP contains various growth factors and cytokines that can reduce inflammation in joint tissues [8]. In our study, EGF levels were not associated with pain-VAS improvement, suggesting that other factors contribute to the observed pain relief. The exact mechanism remains unclear, and further research is needed to elucidate the pathways involved.

There is still controversy regarding the efficacy of PRP intra-articular injection for hip OA. Although there have been some studies comparing the efficacy of intra-articular injection of PRP and HA, there is no consensus. Dallari et al. reported that, at 2, 6, and 12 months, the patients of the PRP group exhibited significantly lower VAS scores compared to those in the HA group, and other scores, including WOMAC, exhibited significant improvement at 2 and 6 months [13]. In addition, Kraeutler et al. reported that the patients of the PRP group had significantly lower rates of conversion to THA compared to HA [14]. On the other hand, Sante et al. reported that the pain-relieving effects of PRP were short term and did not exhibit a significant difference compared to HA [15]. In recent systematic reviews as well, the pain inhibition effect of PRP exhibited no significant difference from that of HA, and its superiority over other intra-articular injections such as HA is still unclear [16–18]. Differences in the PRP preparation method, follow-up duration, patient selection criteria, and OA grades of the patients in the respective studies have been proposed as causes for this variability.

Our study differs from previous reports in that we focused exclusively on patients with hip OA secondary to DDH and used a highly concentrated LP-PRP. The PRP used in this study had a high concentration at 20-fold and was an LP-PRP close to pure-PRP, largely free of leucocytes; the patients had comparatively mild OA and low BMI, and the follow-up duration was short. These factors are conceivable reasons why the pain-relieving effects of PRP were relatively high in our study.

Various surgical procedures have been proposed to alter the mechanical environment of DDH and prevent the early onset of secondary OA in adolescents and young adults. PAO and RAO are widely performed surgeries for DDH, and good results have been reported [4, 5]. In patients who are unable to undergo immediate joint-preserving surgery due to personal or medical reasons, intra-articular PRP injections may offer temporary pain relief and improve quality of life. This could be particularly beneficial as a time-saving measure until definitive surgical intervention is feasible. However, we acknowledge that delaying necessary corrective surgery could lead to further joint deterioration, including irreversible cartilage injury and labral tears [19]. Therefore, PRP injections should be considered with caution and in the context of a comprehensive treatment plan that prioritizes timely surgical intervention when appropriate.

This study has major limitations that warrant emphasis. First, there was no true control arm (i.e. a saline placebo), making it difficult to draw definitive conclusions about the specific effects of PRP. Indeed, some reports have shown that intra-articular saline injections can achieve pain inhibition and functional recovery comparable to those of other medications [20], indicating the need for a future RCT with a placebo. Second, our sample size was small, which may limit the statistical power to detect significant differences. Third, there was an imbalance in baseline factors between the PRP and HA groups—such as the proportion of patients with advanced K–L grades—that could confound our interpretation of the results. Moreover, the use of HA as a standard of care remains controversial, as some studies suggest that its effects are not superior to those of saline injections in the knee [20]. From a purely scientific perspective, comparing PRP to sterile saline in the hip would be highly informative. Furthermore, although intra-articular injections may offer temporary symptom relief, they should not supplant necessary surgical interventions in younger patients with DDH. Delaying surgical treatment could adversely affect long-term outcomes.

Finally, the short follow-up period (24 weeks) may not capture the full duration of the therapeutic effects or potential long-term complications. In the future, a large-scale, long-term RCT is warranted to more conclusively evaluate the efficacy and safety of PRP injections in patients with hip OA and DDH.

## Conclusions

The study finds that PRP treatment is slightly more effective or at least as effective as HA treatment in reducing hip pain for OA secondary to DDH. Treatment with PRP showed notably better pain-VAS scores compared to HA, highlighting its potential. However, similar WOMAC-pain scores between PRP and HA suggest equivalent overall pain management. Therefore, intra-articular PRP injections are a viable alternative to HA for OA secondary to DDH.

## Data Availability

The data underlying this article will be shared on reasonable request to the corresponding author.
